# A Cross-Sectional Survey of Medical Cannabis Users: Patterns of Use and Perceived Efficacy

**DOI:** 10.1089/can.2016.0007

**Published:** 2016-06-01

**Authors:** Michelle Sexton, Carrie Cuttler, John S. Finnell, Laurie K. Mischley

**Affiliations:** ^1^ Department of Medical Research, Center for the Study of Cannabis and Social Policy, Seattle, Washington.; ^2^ Department of Psychology, Washington State University, Pullman, Washington.; ^3^ Graduate School of Integrative Medicine, AOMA, Austin, Texas.; ^4^ Research Institute, Bastyr University, Seattle, Washington.

**Keywords:** Cannabis, health effects, medical Cannabis, survey, use patterns

## Abstract

**Background:** The political climate around Cannabis as a medicine is rapidly changing. Legislators are adopting policies regarding appropriate medical applications, while the paucity of research may make policy decisions around conditions for which Cannabis is an effective medicine difficult.

**Methods:** An anonymous online survey was developed to query medical Cannabis users about the conditions they use Cannabis to treat, their use patterns, perception of efficacy, and physical and mental health. Participants were recruited through social media and Cannabis dispensaries in Washington State.

**Results:** A total of 1429 participants identified as medical Cannabis users. The most frequently reported conditions for which they used Cannabis were pain (61.2%), anxiety (58.1%), depression (50.3%), headache/migraine (35.5%), nausea (27.4%), and muscle spasticity (18.4%). On average, participants reported an 86% reduction in symptoms as a result of Cannabis use; 59.8% of medical users reported using Cannabis as an alternative to pharmaceutical prescriptions. Global health scores were on par with the general population for mental health and physical health.

**Conclusions:** While patient-reported outcomes favor strong efficacy for a broad range of symptoms, many medical users are using Cannabis without physician supervision and for conditions for which there is no formal research to support the use of Cannabis (e.g., depression and anxiety). Future research and public policy should attempt to reduce the incongruence between approved and actual use.

## Background

Cannabis has been used medicinally for a variety of ailments for millennia.^1^ The legalization of medical marijuana in the United States began in 1996, expanding to 25 States and the District of Columbia.^2^ Although medical use has become increasingly common, scholarly reports on locally accessed over-the-counter Cannabis are sparse.

Research on the therapeutic potential of Cannabis has been significantly hindered by Schedule 1 status and by the National Institute on Drug Abuse policy on the legal supply of Cannabis for research. Nevertheless, mounting evidence from controlled studies has provided evidence for the efficacy of Cannabis in the treatment of some medical conditions and symptoms. Specifically, controlled studies have revealed that cannabinoids demonstrate therapeutic potential as an analgesic, antiemtic and appetite stimulant; for Tourette's syndrome, post-traumatic stress disorder, multiple sclerosis, epilepsy, movement disorders, glaucoma, and headaches.^3–8^ However, the external validity of these studies is limited by small sample size, oral administration of Cannabis extracts, administration of isolated cannabinoids, and lack of patient-reported outcomes (PROs). These studies may not represent outcomes for patients using locally accessed inhaled Cannabis or adequately address the potential for cannabis to simply improve quality of life (QOL).

States that have approved medical Cannabis typically define diagnoses for which doctors can recommend Cannabis. Commonly accepted conditions include pain, multiple sclerosis, nausea, spasms, seizures, or other chronic and debilitating conditions.^9^ Despite restrictions on which diagnoses are sanctioned for medical use, clinical experience demonstrates that patients often report relief from a wide variety of symptoms or diagnoses. These patients may also be using Cannabis alongside or in lieu of prescription drugs. It is largely unknown what is the perceived efficacy or effect on global functioning and well-being. The goal of this study was to collect epidemiologic data to inform medical practice, research, and policy, as well as to provoke discussion about the discrepancies between medico-legal recommendations and PROs.

## Methods

### Survey

The Internal Review Board at Bastyr University approved the protocol. Procedures were in accord with the ethical standards of the Helsinki Declaration, as revised in 2008. A literature review was conducted to identify existing epidemiological surveys of Cannabis use.^10–15^ The authors developed a novel questionnaire by assessing strengths and weaknesses of existing surveys and to meet the goals of this study. Drafts were circulated to physician researchers and Cannabis users for feedback in an iterative process. The final survey consisted of 44 structured questions answered by yes/no, multiple choice responses, and rating scales.^16^ These included PROs using the PROMIS^®^ Global Health 10-item short form (part of a National Institutes of Health initiative to produce validated, self-reported item banks for physical, mental, emotional, and social health) to measure overall well-being. Three additional open-ended questions were included in the survey. Study data were collected and managed using Research Electronic Data Capture (REDcap), a secure tool allowing participants to directly enter responses.^17^

Subjects were a self-selected convenience sample who accessed the survey through links posted on the Center for the Study of Cannabis and Social Policy and Bastyr University websites, a Facebook page, flyers in Washington State Cannabis dispensaries, or word of mouth from December 2013 to January 2016. The only inclusion criterion was having used Cannabis at least once in the past 90 days. Twenty-five respondents of a total of 2459 were deemed ineligible and excluded based on this criterion. To minimize risk to participants, no identifying information was collected. Individuals were given the opportunity to provide a five-digit code that enabled repeat responders to be identified with only the first response analyzed. A total of 30 repeat responders were identified and deleted from the total of 2434 eligible respondents. Individuals were told they could skip any question(s) they did not wish to answer. Those who refused to provide a five-digit code are included in the database based on the rationale that fear of lost anonymity is more likely to motivate response refusal than repeat participation.

### Statistical analyses

Descriptive statistics, including means, standard deviations, confidence intervals (CIs), and simple percentages were used to describe demographics, health characteristics, conditions, perceived efficacy, and Cannabis use preferences.

PROMIS scores were calculated using the recommended scoring method that calibrates each score to a US national mean of 50 and standard deviation (SD) of 10.^18^ When calculating T scores, all respondents who skipped any of the items were eliminated. IBM SPSS 23 was used to perform the statistical analyses. Prism Version 6 (GraphPad™ Software, La Jolla, CA) was used to generate the figure.

## Results

### Demographics

One thousand four hundred twenty-nine respondents (of a total of 2404 eligible respondents) opted to identify as medical rather than recreational Cannabis users. Age range was 15–80 years (*M*=36.3, SD=14). Respondents came from 18 countries, with the United States (77.8%), United Kingdom (5.6%), and Canada (3.1%) being the most represented. Only 39.7% of medical users reported obtaining a recommendation from a licensed medical provider. Table 1 displays the remaining demographics of this sample.

**Table 1.** **Demographic Characteristics of Medical Cannabis Users**

Gender	*n* (%)	Missing
Female	644 (45.4)	11
Male	774 (54.6)	
Income: last 12 months		
<$20,000	333 (23.3)	44
$20–40,000	334 (24.1)	
$40–60,000	238 (17.2)	
$60–80,000	151 (10.6)	
$80–100,000	112 (8.1)	
$100–150,000	117 (8.4)	
>$150,000	100 (7.2)	
Highest level of education		
<8th grade	4 (0.3)	10
Grade 9–11	40 (2.8)	
High school/GED	398 (28.0)	
Technical school	198 (14.0)	
Associate	245 (17.3)	
Bachelors	372 (26.2)	
Masters	111 (7.8)	
Doctorate	51 (3.6)	
Age (years)		
15–20	122 (8.6)	16
21–30	498 (35.2)	
31–45	421 (29.8)	
46–60	268 (19.0)	
61–75	103 (7.3)	
>75	1 (0.1)	
Current employment		
Full-time	674 (47.7)	15
Part-time	298 (21.1)	
Unemployed	164 (11.6)	
Retired	78 (5.5)	
Disabled	200 (14.1)	
Ethnicity		
Caucasian	1221 (86.5)	17
Black	12 (0.8)	
Hispanic	46 (3.3)	
Native American	21 (1.5)	
Asian/Pacific Islander	21 (1.5)	
Other	91 (6.4)	

GED, Graduate Equivalency Diploma.

### Conditions

Participants were asked, “Do you use Cannabis for the management of any of the following conditions?” A list of 19 conditions was provided and more than one condition could be selected. An open-ended question for identifying other was also included. The five conditions most frequently selected were pain (61.2%), anxiety (58.1%), depression (50.3%), headache/migraine (35.5%), and other (34.2%). The percentages of individuals using Cannabis for each of the 19 conditions are displayed in Table 2.

**Table 2.** **Total Number and Percentages of Medical Cannabis Users Reporting Use for Each Medical Condition**

Condition reported	*n* (%)
Pain	874 (61.2)
Anxiety	830 (58.1)
Depression	719 (50.3)
Headache/migraine	507 (35.5)
Other	488 (34.1)
Nausea	392 (27.4)
Muscle spasticity	263 (18.4)
Arthritis	245 (17.1)
Irritable bowel	211 (14.8)
Intractable pain	164 (11.5)
Anorexia	142 (9.9)
Cancer	47 (3.3)
Ulcerative colitis/Crohn's disease	45 (3.1)
Other seizure disorder	37 (2.6)
Tics	36 (2.5)
Tremor	33 (2.3)
Glaucoma	25 (1.7)
Epilepsy	18 (1.3)
Multiple sclerosis	16 (1.1)
HIV	10 (0.7)

### Patient-reported outcomes

**FIG. 1.**
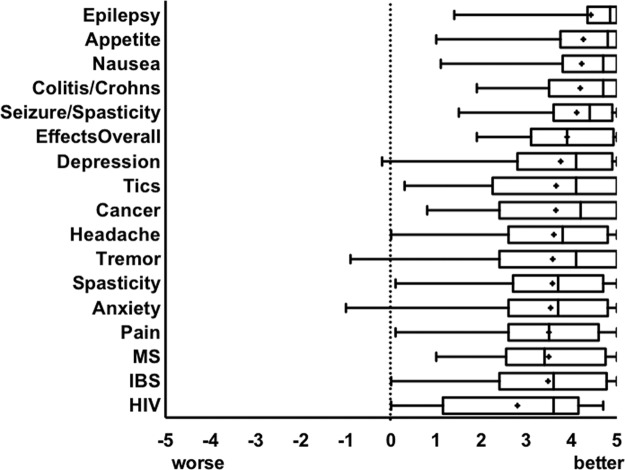
Patient-reported outcomes: participants subjectively scored change in symptoms on a scale of −5 (worsening of symptom) to +5 (symptom improvement). Depicted is the distribution with median. + denotes the mean.

To provide a subjective view of Cannabis efficacy for symptom relief, participants were asked, “Overall, how does Cannabis affect the symptoms you associate with xxx?” (xxx referring to conditions listed in Table 2). Summary of effects was assessed on a scale ranging from −5 (worsening symptom) to 0 (no change in symptom) to +5 (improvement of symptom). Before analyses, these data were examined for univariate outliers, defined as scores falling more than 3.29 standard deviations (*p*<0.001, two-tailed) away from the mean.^19^ A total of 26 scores were identified as univariate outliers and were replaced with a raw score one unit higher than the nearest nonoutlying value.^19^ The mean effect across all conditions was 3.60 (CI=3.55–3.66), which corresponds to an 86% reported improvement in symptoms. Figure 1 is the distribution of the data with the median and mean. As shown in the figure, the greatest perceived efficacies were for epilepsy, appetite, nausea, colitis/Crohn's disease, and seizures/spasticity. The mean effectiveness ratings given for each medical condition with 95% CI and ranges are found in the Supplementary Table S1.

### Substitution for prescription drugs

In response to the question “Have you have ever used cannabis as a substitute for prescription drugs?” 59.8% of participants responded yes. When asked which drugs they substitute Cannabis for, over 25% of these participants reported substituting Cannabis for pain medications, including opiates.

### Routes of administration

Participants were asked to indicate “the method I most commonly use” (for administration). Once again, participants could check all that applied. Inhalation was selected by 84.1%, specifically pipes (31.9%), bongs (19.4%), rolled joints/blunts (16.5%), and vaporization devices (16.3%). Other administration routes/methods (other than flower) included concentrates (oil, keif, hash; 6.4%—also typically inhaled), oral (edibles, tinctures, capsules; 8%), topical (0.6%), fresh juice (0.5%), and other (0.4%).

### Quantity

When shown an image of 1 g of Cannabis, compared with the size of a penny, and asked, “How much Cannabis bud or flower do you usually use per week?” 12.3% reported using less than 1 g per week; 20.3% reported using 1–2 g per week; 31.8% of participants reported using between 3–5 g per week of flower material; 26.1% reported consuming ∼7 g (1/4 ounce) per week; 6% reported using one ounce per week (28 g), and 3.4% reported using more than 28 g (1 ounce) per week.

### Daily dose

Participants were asked, “How many hits do you take per smoking session?” The majority of respondents (60.8%) reported using 1–5 hits per session, 21.3% reported using 6–10 hits per session, and 18% reported that they use more than 10 hits per smoking session. Most respondents reported using 1–4 times per day (47.6%), 14.9% were using 5–10 times per day, and 12.2% reported using all day, every day. The remainder of the respondents reported using less than once a day (25.3%).

### Selection criteria

When queried, “In selecting my Cannabis medicine, I consider these to be important factors,” there were 15 available responses and participants could select multiple response options. The top factors medical patients report to be important for selecting their medical Cannabis are smell (47.2%), claims of high delta 9-tetrahydrocannabinol (THC) potency (45.8%), claims of being a hybrid *indica/sativa* species (45.1%), claim of being an *indica* species (43.3%), how the flower looks (size, density of the flower, and/or trichome and shape; 40.2%), claims of high cannabidiol (CBD; 41.2%), claims of being *sativa* species (38.2%), and varietal name (24.1%).

### PROMIS global health

The 10-item short form developed and published by PROMIS was used to arrive at a bottom-line indicator of health status. We included ratings from five primary PROMIS domains (physical function, fatigue, pain, emotional distress, and social health) and general health perceptions. Before analyses, these data were examined for univariate outliers and four outliers were identified and replaced with a raw score equal to the nearest nonoutlying value (since the outlying values were only one unit higher than the nearest nonoutlying value). Mean scores for each of the 10 items are summarized in Table 3 with standard deviations and 95% CI. By summing the physical and mental health scores separately (using only participants with complete data on each subscale), the standard PROMIS raw score to T score conversion allowed for comparing our sample with the general population. The distributions are standardized such that a score of 50 represents the mean for the US general population, with a standard deviation of 10 points. For mental health, our sample scored 49.33 (SD=8.20, CI=48.90–49.76). For physical health, our sample scored 47.83 (SD=8.40, CI=47.39–48.27), placing these medical Cannabis users as average for global mental health and global physical health compared with the general population.

**Table 3.** **PROMIS**^®^ **Global Scores**

	Mean	Standard deviation	95% CI
Physical health items			
General health	3.46	0.99	3.41–3.52
Physical health	3.27	1.00	3.21–3.32
Everyday function	4.51	0.89	4.46–4.55
Pain	3.36	1.00	3.30–3.41
Fatigue	3.66	0.88	3.62–3.71
Mental health items			
QOL	3.70	0.94	3.65–3.75
Mental health	3.66	1.02	3.61–3.72
Social satisfaction	3.56	1.05	3.51–3.61
Social discretionary	3.80	0.92	3.75–3.85
Emotional problems	3.35	1.01	3.30–3.40

Subjects responded to standardized questions that report quality of life and global health in mental and physical domains.

CI, confidence interval; QOL, quality of life.

## Discussion

Despite federal restrictions on accessing Cannabis for therapeutic and research purposes, patients are increasingly seeking Cannabis to treat a broad range of medical conditions and QOL. Data from this study indicate that patients report QOL on par with the general population and a high level of perceived efficacy for most of these conditions. This is one of the largest surveys of medical Cannabis users published to date. This sample is not expected to be representative of the general population as those who found Cannabis to be ineffective or experienced adverse effects would be unlikely to respond to the questionnaire. While the use of PRO instruments captures concepts related to how a patient feels or functions and is useful in measuring treatment benefit, double-blind placebo-controlled trials are needed to validate specific health claims.^20^

The broad spectrum of conditions reported here does not mirror qualifying conditions in Washington State (origin of the questionnaire) or the United States. Despite mental health conditions being excluded as qualifying conditions across the United States, the second and third most common conditions reported here were anxiety and depression.^9^ California and Maryland are the only states where doctors are permitted to recommend Cannabis for other persistent medical conditions. These data, taken together with pre-clinical data on the role of the endocannabinoid system in stress, suggest utility for anxiety and depression and warrant further investigation in controlled trials with human subjects.^21–30^

PROs for mental and physical health scores were on par with the general population. This result is surprising given that 14% of this population identified as disabled, annual income was reported to be below the median, and the medical conditions they reported would be expected to significantly impact global health. One interpretation is that our sample was not very ill and potentially using Cannabis for recreational purposes. Indeed, 50.9% of our medical sample reported using Cannabis for both recreational and medical purposes. However, these data are similar to results previously published in two other cohorts and may reflect the palliative nature of Cannabis.^31,32^

The average score for perceived symptom improvement was 3.60 (CI=3.55–3.66), corresponding to 86%, similar to 68% who reported symptoms being much better in a previous study.^32^ As this was a self-selected convenience sample of medical cannabis users, the authors acknowledge the potential to overestimate the actual efficacy of Cannabis. Additionally, it is unknown whether marketing of medical Cannabis may produce a placebo effect. While these data add to our understanding, conclusions should be drawn from controlled trials.

Nearly 60% of medical users in our survey report substituting Cannabis for prescription medications. This illustrates that Cannabis may function as a harm reduction tool, particularly relevant with regard to current epidemic of prescription opiate-related deaths.^33,34^

Inhalation using flower is the preferred administration route for the majority of medical Cannabis users. Rapid onset of effect with inhalation allows for quick relief, inclusion of the terpenoid fraction, and immediate feedback for patient titration.^35^ Moreover, patients can vaporize Cannabis flower to reduce respiratory risks associated with smoking.^36^ As a recent trend, some States are confining medical Cannabis use to concentrates, oral administration, or to only certain components, while our results indicate that these restrictions may not necessarily best serve patients. Additionally, the safety of highly concentrated preparations (along with excipients such as propylene glycol) has not been well studied.^37^ These results also highlight individual variations in frequency, amount, and dose (number of inhalations) of Cannabis. Self-titration has been demonstrated to be useful in clinical trials where patients experienced optimization of symptom relief at a dose not associated with troublesome adverse effects.^38–40^ While those studies were conducted with a standardized product, the results are similar to those presented here, demonstrating that patients can self-titrate and achieve relief from several symptoms at one time.

It is significant that 41% of patients seek Cannabis enriched in CBD. CBD was isolated in 1940 from wild hemp (before THC in 1964)^41–43^ and preliminary results suggest it to be effective in relieving both anxiety and depression in animal models and in humans.^30,44,45^ It is important to note that research has overwhelmingly represented the effects of THC, while whole plant studies taking the combined effect of CBD are grossly unrepresented in the literature. CBD when taken in combination with THC has been shown to inhibit undesirable side effects attributed to THC.^46,47^ Differences in effects observed by patients may also be attributed to the Cannabis terpenoid fraction relevant for the ongoing debate by botanists regarding the species designations of indica and sativa and the meaning of these terms for patients.^48,49^ Because various phytochemical classes contribute to overall effects, there is a need for investigation into the synergy of compounds and how they contribute to effect and side effect profiles.^50,51^ Phytochemical biodiversity reflected by the presence of CBD in Cannabis flower is important to patients, particularly because of the suggested anxiolytic potential for those suffering from anxiety and/or depression.^30^

The participants in this survey were a self-selected convenience sample and may not represent all patients who use medical Cannabis. As such, generalizations to global populations of medical Cannabis users should be made very cautiously. Indeed, the results from this survey should be used to inform controlled research rather than to reach definitive conclusions. Limitations of this study include selection bias, self-reporting, exaggeration of perceived efficacy, placebo effects, and recall bias. The sample is largely limited to people who access the internet and are skilled in the use of online tools. Our population also lacks ethnic diversity. This may be partially due to the channels through which the survey was distributed and the origin in Washington State.^52^ It is important to note that African Americans and Latinos are significantly more likely to be arrested for marijuana than Caucasians.^53,54^ The survey may not have reached these populations or they may be hesitant to participate.

We further acknowledge that patients with multiple sclerosis, HIV, cancer, Parkinson's disease, and epilepsy are highly underrepresented in our sample. This is unfortunate given that these are conditions medical Cannabis States recognize as qualifying conditions. Moreover, the underrepresentation of other intractable qualifying conditions obscures comparisons for Cannabis efficacy in the treatment of those conditions relative to the treatment of nonqualifying conditions that were better represented in the survey (e.g., depression and anxiety). Future recruitment strategies will aim to target these important underrepresented patient populations.

Notably, this study had almost equal gender representation. This distinguishes our study from others where male users were the majority^10,12,15,33^; 50% of our population earned less than $40,000 per year, whereas in other studies, 71–73% reported this same income.^10,15^ There is a significant representation here of those with higher education, 37% compared with 18%, 14.5% in other surveys.^55,56^ These data illustrate a changing demographic for medical Cannabis use. Demographics further revealed that the general income for the majority of these participants (65.6%) is below the median income in King County, Washington (Seattle: $73,035).^57^ Patients must pay out of pocket for Cannabis, and access at an affordable price is an important factor.

The American Public Health Association calls for a public health approach to regulating legalized Cannabis and considers this a public health priority.^58^ Observational studies such as this one allow for PRO to inform policy and controlled trials on a variety of topics. Given the current nationwide epidemic of prescription opiate and heroine abuse and related deaths, there is a desperate need for opiate alternatives with good efficacy and safer toxicology profiles. Our data indicate that pain patients are experiencing adequate symptom relief from pain as well as symptom relief from comorbid conditions of depression and anxiety. Substitution of Cannabis for prescription opiates or adjunctive use of Cannabis with pain medications is an avenue requiring further exploration.

In conclusion, Cannabis is being used for a wider variety of conditions than traditionally accepted by the scientific community and reported to be effective for some symptoms. PROs can be useful for informing the development of public policy until such time that sufficient controlled trials can be conducted to validate specific claims.

## Supplementary Material

Supplemental data

## Abbreviations Used

CBD: cannabidiol
CIs: confidence intervals
PROs: patient-reported outcomes
QOL: quality of life
THC: tetrahydrocannabinol
