# Supplementary material for: A Cross-Sectional Survey of Medical Cannabis Users: Patterns of Use and Perceived Efficacy
Source: Cannabis Cannabinoid Res. 2016 Jun 1;1(1):131–8. doi: 10.1089/can.2016.0007 (PMC5549439; doi:10.1089/can.2016.0007)
Supplement: Supplemental data [file Supp_Table1.pdf]

Supplementary Data

Supplementary Table S1. Summary of Perceived Efficacy Ratings for Each Medical Condition

Condition	Mean	95% CI	Min.	Max.
Epilepsy	4.43	3.95–4.90	1.4	5
Appetite	4.26	4.10–4.42	1	5
Nausea	4.22	4.12–4.32	1.1	5
Colitis/Crohn's disease	4.19	3.88–4.50	1.5	5
Seizures/spasticity	4.11	3.77–4.45	1.5	5
Overall	3.90	3.52–4.28	1.9	5
Depression	3.76	3.66–3.85	−0.2	5
Tics	3.66	3.18–4.14	0.3	5
Cancer	3.65	3.21–4.10	0.8	5
Headaches/migraines	3.61	3.50–3.72	0.0	5
Tremor	3.58	3.00–4.16	−0.9	5
Spasticity	3.58	3.43–3.72	0.1	5
Anxiety	3.53	3.44–3.63	−1.0	5
Pain	3.50	3.42–3.57	0.1	5
Multiple sclerosis	3.49	2.82–4.17	1.0	5
Irritable bowel syndrome	3.48	3.29–3.66	0.0	5
HIV	2.80	1.45–4.15	0.0	4.7
Glaucoma	1.50	—[a]	−0.1	3.10

Subjects used a slider bar for effect scale ranging from −5 to +5, with −5 representing worsening symptoms, 0 representing no change in symptoms, and +5 representing improving symptoms.

[a]Insufficient power to compute CI for glaucoma ($n = 2$).

CI, confidence interval.
